# Philadelphia Academy of Surgery

**Published:** 1881-04

**Authors:** 


### PHILADELPHIA ACADEMY OF SURGERY.

*Complete Osseous Anchylosis of the Knee Treated by Removing a Wedge of Bone.*—Dr. Thomas G. Morton presented the following notes of the case:

About two years ago, the patient, John J. B, æt. 15, while chopping wood, accidentally wounded himself in the right knee with the axe, the blade entering deeply just to the right of patella, opening the joint. As a result, anchylosis took place; the limb assumed the position of flexion of leg towards thigh, and finally the bony anchylosis became complete, the function and original structure of the joint being completely abolished.

When admitted into the Pennsylvania Hospital, March 9, 1880, the limb presented the following appearance: The entire lower extremity has undergone considerable muscular atrophy; motions of hip and ankle-joint normal. The leg is flexed at knee to rather more than a right angle, and the anchylosis of the joint is absolute. The patella appears to be slightly displaced outwardly, and is firmly adherent and immovable. The hamstring tendons are very tense. The general condition of the patient is excellent. Kidneys normal.

On March 12, after etherization, I excised with the saw a wedge shaped piece of bone, the base of which included the patella and was two and a half inches in width. It was so cut as to make the wedge a truncated one at its apex, so that when removed, and the sawn surfaces brought into close contact, the leg would be brought not quite straight, and in a slightly flexed position; the patient thus being enabled, after recovery, to walk upon the ball of the foot, obviating the necessity for walking in a stamping manner upon a leg brought out perfectly straight.

After the ligation of a few articular vessels, the flap, which was a large, anterior U-shaped one, was approximated with interrupted sutures of silver wire, a dressing of carbolized charpie was applied, and the limb placed upon a double-inclined plane of very slight degree of inclination, so that the sawn surfaces lay in exact apposition. Prescribed iron and quinia and solution of acetate of ammonia.

March 20.—The patient undoubtedly has erysipelas. The flaps very much swollen. Four stitches removed. Very free discharge of pus. Dressed with ung. zinci oxid. Temperature, 103.5°. Coincident with the rise in temperature a small amount of albumen appeared in the urine, but no casts were found.

March 29.—Albumen has disappeared. Temp. 99°. Erysipelas has subsided, but pus has burrowed back upon the outer side of thigh. This opened, and drainage-tube inserted.

April 6.—Tinct. ferri chlor stopped. Wounds, which now look well, dressed with carbolized charpie.

April 15.—Stopped quinia. The opening in thigh has closed; the line of flap granulating well.

April 26.—Some union exists between the bony surfaces.

May 18.—Granulations fungous. Applied sol. cupri sulph. gr. iv to f℥j.

May 23.—Bony union quite firm. Line of flap almost healed. Leg removed from double inclined fracture-box and laid upon pillow.

June 4.—Plaster dressing applied, and, when set, cut and reapplied. Wound entirely healed.

June 20.—Walking with assistance of crutches.

July 2.—The operation was a success, and the result a perfect one. The limb is in position of slight flexion at the knee, and when he stands erect the ball of the right foot comes to within two and a half inches of the ground. The bony union is very strong, there being no motion at all at the seat of operation. He was ordered a brace, consisting of a shoe with thick sole and high heel, side-irons, and knee and thigh brace.

*The Treatment of Fracture of the Lower End of the Radius.*—Dr. R. J. Levis exhibited three patients under treatment for fracture of the lower end of the radius. These fractures had been produced in the usual way, by falls in which the weight of the body is received on the palm of the hand. Each of these cases had shown in a marked manner, at the time of the receipt of the injury, the ordinary deformity of the fracture. He presented the cases, at from about a week to three weeks from the receipt of the injury, to show the effect of complete reduction of the fracture and maintaining apposition in his moulded splint. The patients were entirely free from pain, and when the splints were removed for examination of the parts, there was good control of the movements of the wrist and hand, and no evidence existed of any of the unfortunate sequences which, it is admitted, generally follow the fracture.

Dr. Levis remarked that, from extended practical observation and the examination of pathological specimens, the following deductions may be summarized:

The ordinary fractures of the lower end of the radius, produced by falls on the extended palm of the hand, are situated at from one quarter to three quarters of an inch above the articular surface, and are transverse in direction. The characteristic deformity of the fracture, as originally described by Colles, is correct, but he erred in locating it an inch and a half above the carpal extremity of the bone.

The theory of the fracture as described by Barton, "a quite small fragment broken from the end of the radius on its dorsal side," has not been verified by clinical experience or by pathological observation, and is not in accordance with the mechanism of the true production of the fracture.

The force which produces the fracture is, for the most part, transverse to the long axis of the bone, and tends only to produce transverse fracture. Violent over extension of the hand is the important factor in its production, and the bone breaks immediately above its strong ligamentous connection with the wrist and hand. Force transmitted through the anterior carpal ligament is the immediate cause of the fracture.

Impaction, to a small extent, may occur by the posterior edge of the upper fragment being driven into the cancellated structure of the lower fragment, but it is not an important complication, and should not prevent coaptation.

Comminution is usually vertical splitting, and is caused by the same mechanical action as that which tends to produce impaction.

The displacement of the lower fragment backward and upward can always be overcome by strong longitudinal traction associated with forced flexion; and, in uncomplicated cases, the fragments, when completely reduced, will remain in apposition without any retentive apparatus.

When comminution by vertical splitting exists, and the fracture has been produced by great force, rupturing the surrounding dense structures, apposition may usually be maintained by keeping the wrist in a state of flexion, with the aid, sometimes, of the pressure of a dorsal pad.

The unfortunate sequences of the fracture, as generally treated, are due to *imperfect primary reduction of the displacement and the want of proper retention in apposition.* The usual long-continued impairment of function of the wrist and hand, and the painfulness which generally follows, are not due, as asserted by most authorities, to inflammation in the sheaths of the tendons, but *simply to pressure and irritation caused by the unreduced fragments.* That such impairment of function and suffering result from the ordinary incorrect treatment of the fracture by surgeons generally, nearly all surgical authorities attest.

In support of this statement Dr. Levis quoted the opinions of Hamilton, Gross, R. W. Smith, of Dublin, Bryant, J. R. Barton, Callender, and Agnew.

Dr. Levis said that many years ago, whilst he was endeavoring to investigate the cause of the usual bad sequences of the fracture under consideration, he recognized its almost uniform position and direction, and concluded that its transverse direction, at that, the thickest part of the bone, could not be produced by longitudinal force, as had generally been taught. Examination of a number of cases of chronic deformity following the fracture, and of many museum specimens, demonstrated the fact that the mechanism of the fracture had not in the treatment of those cases been understood, and that the fragments had never been brought to proper apposition, and thus deformity, with its attendants of more or less suffering and disability, had continued through the lives of the patients.

He did not deny that fracture of the lower end of the radius may be produced by varied direct and indirect forces, and consequently show as much variety in its mechanism. But the form of fracture which is of most frequent occurrence, presenting the familiar characteristics, and which in its results, as ordinarily treated, is truly the opprobrium of the surgery of fractures, is a simple transverse fracture, very near to the carpal end of the bone, can readily be placed in apposition, and should not be followed by deformity and permanent disability. He had clinically taught and practiced on these principles since the year 1861, and had asserted them in address before the Medical Society of the State of Pennsylvania in 1874, and again in a paper before the same body in 1879.

The usual errors in the treatment of this fracture are in not recognizing the position and direction and the peculiar displacement of the lower fragment, and in not producing a correct apposition of the fragments. There is also the customary error of endeavoring to treat a fracture of a curved portion of bone by a straight surface of splint, which tends, after a complete reduction to again displace the lower fragment backwards. A pad fitted to the anterior radical curvature may be placed on a straight splint, but it is liable to slip out of position, and its action to be the reverse of what it is intended to accomplish. The moulded splint has the merit of following the curve of the anterior surface of the lower end of the radius, and the imbedding of the wrist and hand in corresponding portions of the splint secures the curve in its proper position with reference to the fracture.

The fixation of the wrist and hand in a state of slight flexion obviates the tendency of the lower fragment to upward and backward displacement. The moulded splint is of flexible metal, so as to be comfortable to any fleshy or attenuated forearm. It is supplied by all the surgical instrument-makers at a cost of one dollar.

Dr. John H. Packard agreed with Dr. Levis in regard to the importance of perfect reduction, and showed a specimen of fracture of both radii. taken from an old woman, who died from other injuries about five days after admission to the Episcopal Hospital. When admitted to the institution, she was wearing two slight splints on each forearm, but the fragments had not been replaced. The fact of the non-reduction was very easily demonstrated. Coaptation of the fragments was effected without great difficulty, and mantained by simple means, viz., the application of thin slips of wood, properly shaped, along the radical edge of the anterior surface of each forearm. These were retained in exact position by means of broad slips of adhesive plaster.

Dr. Packard urged that the usual method of procedure, by mere extension, and drawing the hand towards the ulnar side, was ineffectual for reduction. By extension, followed by flexion, and moulding with the surgeon's fingers, the normal contour of the bone could be best restowed; and, in order to maintain it, the concavity of the anterior surface of the radius at its lower part should be preserved.

Bond's splint, as commonly employed, is, in his opinion, a most fruitful source of deformity in these cases. He had used Dr. Levis's tin splint with satisfaction.

Dr. J. E. Mears inquired if there was not a difference between fractures entering the joint and those which were not thus complicated.

Dr. Levis replied that there would be more likelihood of arthristis occurring in the former case, but said the results were usually good if replacement was perfect.

Dr. T. G. Morton thought that immediate reduction under either was of the greatest importance, and the kind of splint of little moment.

Dr. D. H. Agnew believed the mechanism of the fracture to be tension of the anterior radio-carpal ligament, and that the injury could be well treated by the method of Bond, with the hand in the natural position of rest. In his opinion Dr. Levis's plan acted by the extensor tendons being put on the stretch and holding the lower fragment down. He had treated cases in the perfectly straight position with good results. Passive motion should be begun early.

Dr. W. H. Pancoast stated that he was accustomed to use two straight splints with appropriate pads, and believed that the line of fracture was above the ligaments.

*Wounds of the Bladder.*—Dr. Morton presented a specimen of wound of the bladder produced in what was supposed to be either a suicidal attempt or an endeaver to relieve a distended bladder by opening the abdominal wall. The man had been found with an incision in the right side of the abdomen made by a sharp instrument and apparently cut from below upwards. The bladder showed a small wound, and the peritoneum the commencement of inflammation. In the urethra there was found at the autopsy a white bone stud, such as used in fastening shirts. This may have produced retension, and caused the man to endeavor to evacuate the bladder by an incision. No history was obtained, because the patient died on the way to the hospital.

Dr. Morton also showed a specimen of large rupture of the bladder taken from a heavy German, who fell over a stool and struck the abdomen. There was urine extravasated among the intermuscular spaces of the abdominal wall, but not much urine was found in the peritoneal cavity,

Dr. Wm. Hunt referred to an instance of perforation of the bladder by a sharp stick, which entered the perineum near the coccyx and opened the bladder. The wound was in a good position for drainage. The boy was catheterized for a time, but in about five weeks was able to pass his urine by the urethra. He finally recovered.

Dr. S. W. Gross had read of a similar case, where the leg of a stool entered the rectum through the anus and tore open the bladder. The patient recovered. The question of operation in cases of rupture of the bladder comes up for decision in some instances of the injury. In an early edition of his work on the Urinary Organs, Professor S. D. Gross had recommended opening the abdomen and mopping out the peritoneal cavity. Walter, of Pittsburg, Heath and Willitt, of London, have done abdominal section in cases of rupture of the bladder with extravasation into the peritoneal sac. Walter's case recovered; and Mason, of New York, and Walker, of Boston, have successfully performed perineal incision for free drainage in injuries of the bladder.

Dr. R. J. Levis believed it advisable, when urine was extravasated, to make a free opening either in the perineum or the supra-pubic region.

*Fatal Hemorrhage from Perforation of the External Iliac Artery Due to Double Psoas Abscess.*—Dr. R. J. Levis showed the specimen taken from a case, with this history:

Sarah J. T., aged 31 years, colored, states that she had never been a robust woman. Her present trouble began last May with pain in the back, etc. About six weeks ago she was compelled to go to her bed. About four weeks ago an abscess pointed and opened about an inch below the anterior superior spine of the ilium, discharging a large quantity of pus daily. When admitted, she had a long superficial sinus running from the opening towards the pubes, which was opened on a director. Her general condition was bad, and she was very weak and anæmic: hence she was placed on iron, quinine, stimulus and nourishing diet. In spite of the careful attention she received, she lost flesh, and the discharge increased. The anterior superior spinous process of the ilium became denuded of periosteum, and bed-sores made their appearance on the prominent points of the back.

On December 25, 1880, shortly after being dressed, she had a severe hemorrhage coming from the abscess, from which she died in a few minutes.

Post mortem examination showed two large psoas abscesses, filling up both iliac fossæ, the one on the left side discharging into the right through a sinus between the spinous processes of the third and fourth lumbar vertebra.

The iliac vessels were dissected away from their attachments with the surrounding tissues on both sides, and a small ulcerated spot on the coat of the external iliac artery of the left side, about the size of a large goose quill, showed a perforation which had allowed fatal hemorrhage. Both abscesses were filled with coagulated blood.

*Death from Hemorrhage occurring in a Child Fourteen Days Old, due to a Scratch of the Lip.*—Dr. John H. Packard recorded this case, in which a mere scratch, laying bare the cutis vera, possibly produced by the child's own finger-nail, began to bleed, and could not be restrained by astringents and the various means employed. The hemorrhage began when the child was seven days old, and recurred, until death took place at fourteen days.—*Philadelphia Medical Times.*

